# Hospital costs of central line-associated bloodstream infections and cost-effectiveness of closed vs. open infusion containers. *The case of Intensive Care Units in Italy*

**DOI:** 10.1186/1478-7547-8-8

**Published:** 2010-05-10

**Authors:** Rosanna Tarricone, Aleksandra Torbica, Fabio Franzetti, Victor D Rosenthal

**Affiliations:** 1 CERGAS-Bocconi University, Via Roentgen 1, 21036 Milan, Italy; 2 Sacco Hospital, Milan, Italy; 3 Medical College of Buenos Aires, Buenos Aires, Argentina

## Abstract

**Objectives:**

The aim was to evaluate direct health care costs of central line-associated bloodstream infections (CLABSI) and to calculate the cost-effectiveness ratio of closed fully collapsible plastic intravenous infusion containers vs. open (glass) infusion containers.

**Methods:**

A two-year, prospective case-control study was undertaken in four intensive care units in an Italian teaching hospital. Patients with CLABSI (cases) and patients without CLABSI (controls) were matched for admission departments, gender, age, and average severity of illness score. Costs were estimated according to micro-costing approach. In the cost effectiveness analysis, the cost component was assessed as the difference between production costs while effectiveness was measured by CLABSI rate (number of CLABSI per 1000 central line days) associated with the two infusion containers.

**Results:**

A total of 43 cases of CLABSI were compared with 97 matched controls. The mean age of cases and controls was 62.1 and 66.6 years, respectively (p = 0.143); 56% of the cases and 57% of the controls were females (p = 0.922). The mean length of stay of cases and controls was 17.41 and 8.55 days, respectively (p < 0.001). Overall, the mean total costs of patients with and without CLABSI were € 18,241 and € 9,087, respectively (p < 0.001). On average, the extra cost for drugs was € 843 (p < 0.001), for supplies € 133 (p = 0.116), for lab tests € 171 (p < 0.001), and for specialist visits € 15 (p = 0.019). The mean extra cost for hospital stay (overhead) was € 7,180 (p < 0.001). The closed infusion container was a dominant strategy. It resulted in lower CLABSI rates (3.5 vs. 8.2 CLABSIs per 1000 central line days for closed vs. open infusion container) without any significant difference in total production costs. The higher acquisition cost of the closed infusion container was offset by savings incurred in other phases of production, especially waste management.

**Conclusions:**

CLABSI results in considerable and significant increase in utilization of hospital resources. Use of innovative technologies such as closed infusion containers can significantly reduce the incidence of healthcare acquired infection without posing additional burden on hospital budgets.

## Background

Considering the rapid pace of innovation in the healthcare arena, an ever-increasing number of strategies for detection, prevention and treatment of diseases are expected in the market. However, budgetary constraints always make it more challenging for policy makers to finance technological innovation in healthcare. Identifying the optimal allocation of available resources in order to maximize health gains in the patient population is a continuous challenge to health-care system sustainability. The dilemma of whether to invest in a new technology or expand existing program to a wider target population is universal. In making those judgments, decision makers apply differing criteria and rely on various sources of information. Economic evaluation analysis, together with assessment of clinical effectiveness, supports decision making processes in public domain by providing necessary information concerning the economic aspects of resource absorption by different healthcare technologies.

Healthcare-associated infections (HAIs) are one of the most serious patient safety issues in healthcare today, affecting over 1.4 million people worldwide (Global Patient Safety Challenge, 2005-2006, World Health Organization). Even though the principal risk factors and appropriate prevention methods have been identified in the past decades, HAIs continue to present one of the major public health problems in the world [1]. Sound and abundant evidence demonstrates that HAIs are associated with increases in morbidity and mortality, as well as greater costs of hospitalization and overall medical care [2-9].

In the United States, the incidence of HAIs has been estimated at 2 million cases per annum, causing approximately 90,000 deaths and imposing an annual financial burden of 6.5 billion dollars [1,10]. In England, it is estimated that about 320,000 patients acquire one or more infections during hospitalization per annum, costing the National Health Service as much as £1 billion a year [11]. In Italy, every year 450,000-700,000 patients acquire infections while in the hospital; in other words, 5 to 8 of 100 hospitalized patients contract a HAI. A few studies have estimated the clinical burden of HAIs, but the evidence regarding the economic impact is currently very limited [7,12]. It was estimated that the economic burden of these infections is equal to 1.0% of total National Health Service expenditure [5,7]. Zotti and colleagues prevalence of HAI was 7.84%, with marked differences among the participating hospitals (range: 0-47.8%). The authors concluded that patients with HAI on average experience longer hospital lengths of stay. Nevertheless, no data was provided in support of that conclusion. Another study investigated the longer hospital stay and extra direct costs of all hospital-acquired laboratory confirmed bacteremia in a 2000-bed teaching hospital. The results showed that HAIs prolonged hospital stay by approximately 20 days and increased direct costs by € 16,536 per case [7].

The highest rate of majority of HAIs occurs in intensive care units (ICUs), and most are associated with the presence of invasive devices such as a central line (CL) or mechanical ventilator [13]. Several million intravascular devices are purchased each year by hospitals and clinics as they are indispensable for administering lifesaving therapies to critically ill patients. However, their use may put patients at risk of local and systematic infectious complications, including both localized site infections and central line-associated bloodstream infections (CLABSI). Nearly 1 of 4 catheterized patients with a central line in place for an average of 8 days is expected to develop catheter colonization, which increases the risk of more serious bacteremia [14,15]. Rosenthal et al showed that ventilator-associated pneumonia and CLABSI represented more than 70% of all device-associated infections in 55 ICUs in 8 countries (41% and 30%, respectively) [16].

CLABSI infections not only complicate illness, but can lead to disability and even death. The mortality attributable to CLABSI was estimated to range between 12 to 25% in several studies [17-20]. In addition, there is a considerable amount of evidence demonstrating that CLABSIs are associated with significant increases in the length of hospital stay and medical care costs [2,8,16,21-25]. Numerous strategies have been evaluated to reduce the clinical and economic burden of CLABSI, such as the use of silver or antiseptic impregnated catheters, cutaneous antisepsis and antimicrobial lock solutions [26]. There is growing evidence that implementation of a "bundle" of multiple interventions can markedly reduce rates of CLABSI [27]. These bundles may include both behavioral (e.g., maximal sterile barrier precautions, catheter placement and optimal timing of replacement, surveillance, education, improved hand hygiene [HH] technique and compliance, etc.), and technological (e.g., use of preferred skin antiseptics such as chlorhexidine gluconate, closed infusion containers, catheter dressings, etc.) practices. Catheter audit programs have also been used to review clinical practice associated with the insertion and subsequent care of CLs and their possible relationship to the development of HAI [28].

The use of innovative, "closed" infusion container has shown to have remarkable impact in reducing the incidence of CLABSI [29]. Closed infusion containers consist of fully collapsible plastic containers that do not require or use any external vent (air filter or needle) to empty the solution, and have injection ports that are self-sealing. Alternatively, the traditional open infusion container consists of rigid (glass, burette) or semi rigid plastic containers that must admit air to empty (air filter or needle) [16]. The risk of contamination and administration-related BSI is increased with open infusion containers that permit air, and potentially microorganisms, to enter. Innovative closed infusion containers have been developed to reduce this risk.

While open infusion containers have been used worldwide for over 75 years, they have been supplanted by closed containers throughout North America and Western Europe. Open containers are still widely used in Latin America, Asia, Eastern Europe, Germany and Italy. Italy is one of the few Western European countries that mainly use open, externally vented glass or semi-rigid infusion containers. At present, there is no empirical evidence available regarding the economic and clinical impact of the introduction of closed infusion containers into clinical practice in Italy.

In order to allow hospital managers to identify the most convenient strategies for reducing the impact of HAIs, it is important to provide reliable data on the costs borne by the hospital for CLABSI and on the cost opportunity to implement an innovative technology aimed at reducing the burden. The present study was designed to meet these two objectives: (1) to measure and evaluate the direct health care costs of CLABSI and (2) to calculate the cost-effectiveness ratio of the closed vs. open infusion container in a hospital setting.

## Methods

### Study design

In order to measure the direct health care costs of CLABSIs, a case-control study was performed in a 500-bed teaching hospital Sacco in Milan, Italy. Table 1 reports data about the size of the hospital and its activities in comparison to other public hospitals in the Lombardy region and in Italy. The Sacco Hospital can be considered representative of other hospitals in the region in terms of size and activity (number of inpatient admissions) and type of patients treated (case-mix) (Table 1).

**Table 1:** Study site characteristics in comparison to all public hospitals in Lombardy and Italy

	Sacco Hospital	Lombardy Region	Italy
Size			
n. of inpatient beds	522	542	289
n. of day hospital beds	66	58.4	34
N. of admissions in 2005			
inpatient	16,322	18,791	10,785
day hospital	1,231	2,783	1,342
Average LOS in 2005	10.6	12.31	11.08
Case mix index	1.11	1.07	1

LOS = length of stay

The cost analysis was conducted alongside a prospective surveillance cohort study aimed at measuring the CLABSI rates of the two infusion containers. The study included four ICUs in the hospital: Coronary (UCC), Post-Acute (TIPO), General (RIA) and Infectious Diseases (ID). The perspective of the analysis was that of the hospital. The detailed methodology of a surveillance study has been published elsewhere [30]. For clarity and completeness, we here shortly present the methods relevant for the economic part of the analysis.

The study was conducted in three sequential phases:

1) Phase 0 (lead-in phase): from November 2003 to February 2004. In this phase, healthcare professionals working in the four ICUs were trained to comply with proper HH and CL care.

2) Phase 1 (open infusion container): from March 2004 to February 2005. In this phase, the current open drug delivery container (glass) was used on all patients admitted to the four ICUs and enrolled in the study.

3) Phase 2 (closed infusion container): from March 2005 to February 2006. In this phase, the innovative, closed drug delivery container (Viaflo^® ^flexible bags) was introduced and used for all patients admitted to the four ICUs and enrolled in the study.

The following controls were implemented to minimize the effect of confounding factors inherent in the sequential comparison design of the study: no new infection control interventions, training programs, products or technologies were introduced during the study periods and all of the investigators, key study personnel, classifications and diagnostics techniques remained constant throughout the entire study. The time effect was mitigated by equal 12-month periods covering all seasons of the year. A lead-in period was performed to standardize HH and CL care compliance practice.

During all phases of the study, active prospective monitoring of HH and CL care compliance (i.e. placement of gauze of CL insertion sites, conditions of gauze dressing - absence of blood, moisture and gross-soiling; occlusive coverage of insertion site - and documentation of date of CL insertion) was conducted and healthcare professionals were regularly informed about their performance. Once the level of compliance set up by the study protocol was achieved (≥ 95% and ≥ 70% for CL care and HH compliance, respectively), the four ICUs could begin to enroll patients (phase 1) [30].

The distinction among the three phases was relevant for the cost-effectiveness part of the economic analysis, since the effectiveness of the two infusion containers was measured in terms of CLABSI rate incurred in the two periods (phase 1 and phase 2), as explained below. As to the cost analysis of CLABSI, it was assumed that the cost of HAI does not vary across phases; therefore, patients for the cost analysis were enrolled throughout the entire study period.

All adult (>18 years of age) patients admitted to the four ICUs with CL in place for the administration of fluids for at least 24 hours were eligible for recruitment. Exclusion criteria included day-hospital patients; patients receiving chronic antibiotics (3 weeks or longer) and presence of other major HAI such as ventilator-associated pneumonia and catheter-associated urinary tract infections.

Data were prospectively collected at admission and included patient demographics (sex, age, and employment status), clinical variables (underlying disease: primary and secondary diagnosis at admission, average severity of illness score [ASIS] [31], type of admission (medical vs. surgical), the placement or removal of CL, number of CL days, presence and type of CLABSI), as well as ICU admission and discharge dates.

Patients who developed CLABSI while in ICUs in the study period were classified as "cases". For multiple admissions and/or multiple infections, only the first ICU admission and/or HAI episode was considered. Patients who did not develop CLABSI at anytime during their stay in any of the four ICUs were eligible to serve as controls, but before being selected they were matched to cases on the basis of the following five variables: 1) sex; 2) age (± 5 years); 3) ASIS (± 1 point); 4) admission department; and 5) type of admission (surgical vs. medical). Patients had to match exactly on all five variables to become controls. Each case was matched with at least 2 controls.

### Resources Consumption and Cost Estimation

Once the cases and controls were identified, further data on consumption of resources were collected and the full costing method was used to evaluate patient admissions. Resources were classified as *direct*, if the consumption was entirely attributable to the patient's hospital stay; *indirect*, if it was difficult to trace the consumption to the patient (e.g., heating, cleaning); and *overhead*, if it was impossible to attribute the consumption to any specific patient (e.g., administration costs). The consumption of resources was measured through a bottom-up approach, by going through each individual patient. *Direct resources *were evaluated in monetary terms through a micro-costing approach, where quantities and unit costs were first estimated and then multiplied); *indirect resources and overheads *were estimated through a gross costing method and were allocated to patients by length of stay.

Data on the quantity and type of direct resources used while in hospital were collected at the patient level through a purposely designed questionnaire (Economic Form) and included: 1) pharmaceuticals including antibiotics; 2) laboratory tests; 3) diagnostic tests; 4) medical procedures; 5) surgical interventions; 6) specialist visits; and 7) medical supplies.

Unit costs for the majority of direct resources were provided by the Accounting Department of the Sacco hospital (points 1, 2, 3, 6 and 7 above). When unit costs were not available (4 and 5 above), the regional tariffs were used. If there were different types of the same resource (i.e., different types of catheters, tubes, etc.), weighted unit costs adjusted by market share were used.

Indirect costs and overheads were calculated at the ICU level and allocated to patients on the basis of their length of stay. These cost categories referred to: department personnel cost including medical doctors, nurses and other professionals; maintenance and equipment repair costs; depreciation costs; administration costs; and hotel costs such as laundry, meals, cleaning, etc.

In the cost-effectiveness analysis, both the direct and indirect components of the production costs of the two infusion containers were evaluated, and the difference was then calculated. A study specific questionnaire was prepared and submitted to the Chief of the Hospital Pharmacy Department. Through the use of the questionnaire, it was possible to identify the cost function of either container in terms of time spent by pharmacists, supplies, wastage, storage, transportation and administration. The unit costs were provided by the Hospital Pharmacy and the Accounting Department. According to the data provided, 1 cent more was applied to the unit cost of the closed container.

Finally, the incremental cost between the two infusion containers was then compared to the incremental effectiveness of the closed vs. open container. The effectiveness was measured in terms of CLABSI rate (number of CLABSI per 1000 CL days in phase 1-open infusion container, vs. phase 2-closed infusion container). In other words, the incremental effectiveness of the closed container was the "number of CLABSI infections avoided" by switching from one infusion container to another.

### Data Analysis

All statistical analyses were performed using the software STATA 9.0 (Stata Corp., College Station, TX, USA). Means with standard deviations were used to describe continuous variables, while medians were calculated for non-normal distributed continuous variables. Clinical and demographic differences between the two groups (cases and controls) were analyzed by performing Student t-test. For categorical variables, a Chi-squared test was used.

The cost of one CLABSI was calculated as the difference in costs between patients who developed CLABSI (cases) and those who did not get infected (controls), after matching for selected variables. A two-sided p value of < 0.05 was deemed to be statistically significant.

A multiple regression model was used to assess the impact of CLABSI on total healthcare costs. More specifically, the regression was preliminarily run by including all variables that were *a priori *believed to be predictors of costs: age, sex, ASIS, department and type of admission. The categorical variables were included in the model as dummy variables. A backward stepwise approach was used, where the model was refined by eliminating coefficients with p values higher than 0.05. Due to its non-normal distribution, the natural log of cost was used as the dependent variable [32,33]. A scenario analysis was performed using different incremental effectiveness rates.

## Results

### Characteristics of the Sample

A total of 1446 patients were enrolled in the study: 273 patients in Phase 0, 608 patients in Phase 1 and 565 patients in Phase 2. The majority of patients were males (67%), with a mean age of 65.5 years. Medical admissions accounted for approximately 75% of hospitalizations.

CLABSI occurred in 43 patients (7, 29 and 7 in three phases respectively); one patient developed two episodes of CLABSI, and the second episode was excluded from the study. Laboratory confirmed bloodstream infection accounted for 18 cases (42%), while the rest of the cases were diagnosed as clinical sepsis. The overall incidence of CLABSI was 6.14 infections per 1000 CL days, and the CL was in place for an average of 6 days per patient. A total of 97 patients were selected as controls (21, 55 and 21 in three phases respectively). The two groups were perfectly comparable; no significant difference was found for age, sex, department, type of admission and ASIS (Table 2).

**Table 2:** Sample characteristics

	Cases (N = 43)	Controls (N = 97)	P value
**Age**			
Mean (std. dev.)	62.1 (16.8)	66.6 (16.2)	0.143
**Gender**			
female	24 (56%)	55 (57%)	0.922
male	19 (44%)	42 (43%)	
**Professional status**			
employed	14 (33%)	22 (23%)	0.319
retired	25 (58%)	69 (71%)	
unemployed	4 (9%)	6 (6%)	
**ASIS**			
1	5 (12%)	19 (20%)	0.397
2	13 (31%)	28 (30%)	
3	7 (17%)	21 (23%)	
4	14 (33%)	18 (19%)	
5	3 (7%)	7 (8%)	
**Department**			
UCC	4 (9%)	10 (10%)	0.897
TIPO	12 (28%)	26 (27%)	
RIA	15 (35%)	39 (40%)	
ID	12 (28%).	22 (23%)	
**Type of admission**			
surgical	3 (7%)	9 (9%)	0.654
medical	40 (93%)	88 (91%)	
**Type of BSI**			
lab confirmed	18 (42%)		
clinical sepsis	25 (58%)		

Test Chi-2 for categorical variables, Student t test for normally distributed data and Mann Whitney U test for ordinal and non-normally distributed (skewed) data.

Cases stayed in the hospital significantly longer than controls; length of stay for cases was approximately two-fold higher than for controls (17.41 days vs. 8.55 days; p < 0.0001). This result varied greatly among different ICUs, ranging from 4.21 extra days in the UCC to 11.09 extra days in the RIA (Table 3).

**Table 3:** Mean (median) length of stay by Intensive Care Unit (days)

Intensive Care Unit	Cases (N = 43)	Controls (N = 97)	Difference in means (days)	P value*
UCC	11.25	7.40	4.21	0.19
(n = 14, 4 cases)	(9.50)	(7.50)		
TIPO	11.66	5.53	6.13	<0.001
(n = 38, 12 cases)	(10.50)	(5.00)		
ID	21.58	12.13	9.45	0.002
(n = 34, 12 cases)	(20.00)	(9.50)		
RIA	20.33	8.84	11.09	<0.001
(n = 54, 15 cases)	(20.00)	(8.00)		
**Overall**	**17.41**	**8.55**	**8.46**	**<0.001**

*Mann Whitney test

Total direct healthcare costs per patient resulted in € 18,241 for cases and € 9,087 for controls. The difference between the two groups was statistically significant in all cost categories with the exception of medical procedures, supplies and surgical interventions, due to the small number of patients receiving them in both groups. The difference was particularly evident for drugs, laboratory test and specialist visits. As to drugs, the total cost for cases was 2.7 times higher than for controls, with almost two-fold costs associated with antibiotics. Laboratory test costs were significantly higher (+180%) for cases as well as cost for specialist visits (+140%). In both groups, length of stay represented the most significant cost component: 78% and 77% of the overall costs for cases and controls, respectively. Because of the greater length of stay for cases, the extra hospital stay cost attributable to CLABSI was € 7,180 (Table 4).

**Table 4:** Unit cost, number of users and mean cost per patient in different cost categories

Cost Category	Average unit cost per category	Total n. of user (% of total sample)	Cost by cases (% of total costs)	Cost by controls (% of total costs)	Δ (Δ/controls)	P value*
Drugs	3.91	139	1,158 (6%)	315 (3%)	843 (2.7)	0.000
Antibiotics	2.76	108	477 (3%)	178 (2%)	299 (1.7)	0.000
Supplies	34.5	140	407 (2%)	274 (3%)	133 (0.5)	0.116
Medical procedures	785.6	38	130 (1%)	301 (3%)	-171(-0.6)	0.899
Surgeries	5968	32	1,728 (9%)	904 (10%)	824 (0.9)	0.607
Diagnostic tests	64.24	137	391 (2%)	232 (3%)	159 (0.7)	0.009
Lab exams	12.4	134	264 (1%)	93 (1%)	171 (1.8)	0.000
Special visits	16.53	55	26 (0%)	11 (0%)	15 (1.4)	0.019
Hospital stay						
TIPO	1140.07		14,137 (78%)	6,957 (77%)	7,180 (1.0)	0.000
ID	350.91					
RIA	1090.68					
UCC	550.11					
**Total Direct Healthcare Costs**			**18,241**	**9,087**	**9,154 (1.0)**	**0.000**

* Student t test on log transformed data

The extra cost attributable to CLABSI was € 9,154 (p < 0.0001) ranging from € 14,757 (p < 0.0001) in the RIA to € 456 (p = 0.1931) in the UCC due to the low number of cases (Table 5).

**Table 5:** Mean (median) cost per patient by Intensive Care Unit (€)

Intensive Care Unit	Cases	Controls	Δ in means	P value*
UCC	7,694	7,238	456	0.193
(n = 14, 4 cases)	(6,703)	(6,965)		
TIPO	19,293	9,119	10,174	<0.001
(n = 38, 12 cases)	(16,842)	(8,721)		
ID	10,479	5,323	5,156	0.002
(n = 34, 12 cases)	(8,747)	(4,416)		
RIA	26,421	11,664	14,757	<0.001
(n = 54, 15 cases)	(24,439)	(10,000)		

* Student t test on log transformed data

These findings were tested in a multiple regression model. The model was robust; it explained almost 50% of total cost variability (R^2 ^= 0.4485). The regression analysis showed that three variables had a significant impact on costs: ASIS, department type and presence of infection. On average, total costs increase by 12.78% per each incremental ASIS grade (coefficient = 0.1278). For CLABSI, the coefficient of 0.673 implies that on average, total hospital costs increase by 67.3% in the presence of this type of HAI (Table 6).

**Table 6:** Multiple Regression Analysis. Dependent variable: Log total cost

Independent Variables	b	T	P value
ASIS	0.1278	2.25	0.026
RIA department	0.6152	4.73	0.000
TIPO department	0.6078	4.72	0.000
CLABSI	0.6736	6.65	0.000

N = 140; p < 0.0000; R = 0.5344; R square = 0.4485

### Cost-Effectiveness of Closed vs. Open Infusion Container

The closed infusion container was more effective than the traditional open container. The number of CLABSI per 1000 CL days in the closed infusion container phase was significantly lower than in the open container phase (3.5 vs. 8.2 p = 0.01). The relative risk (RR) was 0.43 with a 95% confidence interval (CI = 0.22 - 0.84) [30]. Thus, the incremental effectiveness of the closed infusion container was 4.7 CLABSI avoided per 1000 CL days. This result was assessed against the incremental costs in order to calculate the incremental cost-effectiveness ratio.

For the majority of cost components evaluated in the questionnaire, there was no measurable difference between the two infusion containers (Table 7). Management of orders, storage space and transportation from the store room to the department did not differ between the two containers. Storage place was not a scarce resource for the Pharmacy Department which had sufficient room to store either bottles (more voluminous) or plastic bags. In other words, the opportunity cost to store bottles was null for the Pharmacy Department at Sacco hospital. The use of the innovative closed infusion container technology did not have any impact on the transportation of supplies from storage to the hospital departments since the service is outsourced and paid for by Sacco hospital according to predefined fixed hourly fees, which do not vary by the amount and/or weight of supplies transferred. Therefore, no difference could be found between the two containers for storage and transportation costs from the hospital perspective.

**Table 7:** Production costs of the two drug delivery containers (€)

Production Function Phases	Open containers	Closed containers
Supplies, preparation and administration costs *	2.98	3.00
Waste management*	0.185	0.0185
Storage management	No difference	
Transportation to departments	Flat rate	

* the calculation is based on one dose of NaCl solution 100 ml

A small difference was found in the cost of disposables, preparation and administration of the two infusion containers (Table 7). The difference relates to cost of disposables (plastic bag vs. glass bottle, needles, alcohol, swabs, etc.). The time needed to prepare the intravenous drug delivery container was estimated to be equivalent (1.5 minutes) by the Chief of the Pharmacy Department, regardless of the type of container used. The administration time was estimated to be 5 minutes in both cases.

The most relevant difference between the two infusion containers was observed in the management of waste. This difference is directly correlated with the weight of plastic bags vs. glass bottles, which for the same volume of liquid is approximately 10 times heavier for the glass bottles than the plastic bags. The cost of waste management is therefore significantly lower for the closed container (Table 7).

In order to measure the level to which the difference in production cost of the two infusion containers could increase while leaving the hospital cost neutral, a scenario analysis was performed. Two scenarios were envisaged on the basis of the incremental effectiveness of the closed container, corresponding to the lower and upper limit of the 95% confidence interval obtained in the surveillance study (RR = 0.43; 95% CI = 0.22-0.84) [30]. In both scenarios, the calculations were performed for 500 catheterized patients for a total of 3000 CL days (average number of patients and CL days observed in the surveillance study) [30]. The baseline was 8.2 CLABSI per 1000 CL days for the open infusion container phase. In a conservative assumption, only the direct costs of CLABSI are considered to be avoidable in short term. In less conservative assumption, all costs are deemed avoidable in the long run. On the basis of these assumptions, direct costs avoided with the closed container range from € 15.4 to € 75.8 per patient in the worst and the best scenarios, respectively. Thus, hospital costs that can be avoided in the short term range from € 7,770 to € 37,800 for every 500 patients catheterized in the best and the worst scenarios, respectively. In the less conservative, full costing approach, avoided costs range from € 72.0 to € 350.8 per patient, or from approximately € 36,000 to € 175,000 for 500 catheterized patients in the long-run.

These results indicate that the innovative technology allows for avoiding hospital costs even when the incremental effectiveness is at its lowest rate. This suggests that even if the difference in acquisition costs of the two infusion containers had been greater than what was observed at Sacco hospital, the new technology would have remained cost-saving.

## Discussion

From the results of the study, it clearly emerges that even a single CLABSI displaces a relevant amount of hospital resources that could be allocated differently. This study provides one of the most comprehensive estimates to date of the economic burden imposed by CLABSI occurring in adult patients admitted to ICUs in Italy. In this study, patients with CLABSI, on average, incurred hospital costs that were almost two times higher than those without CLABSI. The majority of the additional costs incurred were due to a prolonged hospital stay. The total healthcare cost attributable to CLABSI averaged € 9,000. These results are in line with those reported in the international literature. They are similar, for example, to those conducted in 309 patients with HAIs treated in a district hospital in England [11]. The author of the UK study estimated that CLABSI cases, on average, had an increased ICU length of stay of 4 days, with hospital costs 2.9 times higher than uninfected patients (extra cost of approximately € 10,000). More recently, Warren and colleagues estimated attributable costs of CLABSI among ICU patients in a non-teaching hospital in the United States. The results showed that CLABSI significantly prolonged hospital and ICU length of stay by 7.54 and 2.41 days, respectively, with extra costs of approximately $ 11,971 [25]. Finally, our results are similar to those obtained in a multi-center study conducted in Calgary, Canada where the median cost attributable to ICU-acquired CLABSI was $ 12,321 CA per case [34].

The authors of a recent review on studies investigating the costs of HAIs conclude that available literature presents several methodological limitations. According to the authors, a majority of published studies use crude costing methods, providing only aggregate estimates [1]. Additionally, it may be argued that the for the most part available studies investigated the impact of HAIs in a retrospective design and, therefore, relied greatly on the availability of cost data from the hospital databases.

The present research represents an attempt to overcome some of these limitations. First, it is a methodologically rigorous cost analysis, including not only costs of hospital stay, but also cost of drugs, antibiotics, medical procedures, surgeries, diagnostic tests, lab exams, and specialists' consultations. Second, it is based on prospectively collected data with study specific questionnaire. Furthermore, the micro costing approach allowed the identification of all resources used by each individual patient with CLABSI, in terms of types and quantities. These data may be of value to other hospitals in Italy and elsewhere to assess, after adjusting for the hospital-specific unit costs, the economic burden of the infections in their contexts.

Results confirmed that hospital stay represents the most significant part of the overall costs in both categories of patients, and primarily accounts for the difference in the incremental costs between the two groups of patients (17.9 vs. 8.5 days; p < 0.0001). It is important to underscore that the present research was conducted from the hospital perspective. It is usually the hospital rather than society who serves as the decision maker when it comes to implementing new infection control interventions such as the use of new drug infusion containers. Because of this perspective, the time horizon of the analysis is limited to the hospitalization period. It is arguable, however, that HAIs impose significant burden in other settings as well. Following discharge, patients who suffered a HAI might consult primary and community care services, such as general practitioners. In addition to the costs incurred by the healthcare sector, there may be costs incurred by the patient and informal caregivers. Further analysis could therefore be considered to expand the perspective of this analysis.

There are some limitations to this study that are worth mentioning. First, there may have been confounding variables that could have influenced the magnitude of the findings and for which we did not account. This type of limitation is typical of observational cohort designs. For example, severely ill patients are more likely to remain in the hospital for prolonged periods because of the severity of illness and not because of HAI. In our study, the additional costs attributable to CLABSI were estimated by matching cases to controls, where the total healthcare costs of cases and controls were directly compared and the difference was determined to be the cost of infection. As these two groups may have different characteristics which might impact resource use, patients with CLABSI were matched with two or more uninfected controls. This methodology was criticized as leading to large overestimates for HAI costs due to biases and confounding variables overlooked in the matching process [3]. In order to overcome the limits of matching design, the use of statistical regression analysis was proposed, wherein the impact of each single variable onto total costs was analyzed with other variables being equal. These methods reduce, if not eliminate, the role played by bias and confounding variables [3].

The second objective of the study was to investigate the incremental cost-effectiveness ratio (ICER) of the innovative technology from the perspective of the hospital. Basically, the question was: what is the incremental cost per avoided CLABSI by switching from the open to the closed infusion container? To respond to this question, an incremental analysis was conducted to measure and compare the costs and outcomes of the two containers.

The innovative, closed infusion container was found to be a dominant cost saving strategy as the adoption of this container significantly reduced the rate of CLABSI without increasing hospital costs. Moreover, no measureable cost difference was observed in the production function of the two containers in the management of orders, storage space and transportation from the store room to the departments. Preparation and administration costs were equivalent. The closed container presented a significantly lower cost of waste management. It must be noted that the results obtained in this hospital may not be entirely representative as to production cost function in other settings. First, the acquisition costs of the two containers are not representative of Italian market prices since they were negotiated at special conditions to facilitate the conduct of the study. Second, in other hospitals, it is likely that some cost components may decrease by switching from the open to the closed infusion container (e.g., storage and transportation). Therefore, the dominance of the innovative technology is likely to be further confirmed if not more prominent in those hospitals where the storage and transportation costs do represent an opportunity cost. In addition, given the estimated full cost of infection of approximately € 9,000, the dominance of the closed infusion container would likely be confirmed even at higher acquisition price of this innovative technology. Furthermore, the scenario analysis demonstrated that the dominance of the closed container is maintained even if the clinical effectiveness in preventing infections is reduced.

## Conclusions

Infections acquired in hospital settings impose a significant burden on both patients and hospitals by significantly increasing hospital length of stay and the overall cost of care. Strategies put in place to reduce the incidence of these infections have positively impacted not only patient quality of life but also hospital budgets. The improved clinical effectiveness of closed infusion container in controlling HAIs has already been demonstrated [29]. In times of resource constraints, the incremental benefits of innovative technologies must be weighed against the incremental costs to assess whether innovations are worth the investment. The closed intravenous drug delivery container represents a rare example of innovative healthcare technology that contributes to the improvement of patient health by concurrently reducing healthcare costs. This implies that by either decreasing or preventing HAIs through adoption of closed drug delivery containers, significant hospital resources can be freed for alternative uses.

This article has reported one of the most comprehensive results to date on the cost of CLABSI in Italy. This study makes it possible to estimate the cost of CLABSI in other general hospitals in Italy after adjusting for incidence rate. We believe that the present analysis is not only a novel contribution to currently available scientific evidence regarding the economic impact of hospital infections in Italy, but can also facilitate better informed decisions about the adoption of innovative infusion containers in Italian clinical practice.

## Competing interests

The study was funded by an institutional grant from Baxter Spa, Rome, Italy.

The views expressed in this paper are solely those of the authors who have no conflicts of interest directly relevant to the content of the paper. The sponsor did not have any role in design and conduct of the study; collection, management, analysis, and interpretation of the data, and preparation, review or approval of the manuscript.

## Authors' contributions

RT has been in charge of conception and design of cost and cost-effectiveness analysis. AT has made substantial contribution to acquisition of data, data analysis and interpretation. FF has significantly contributed to the design of the clinical part of the study and has been in charge of data acquisition in the Sacco hospital. VR was the main clinical investigator and has been responsible for the clinical part of the study and clinical data adjudication, validation, collection, uploading and analysis. RT and AT have been involved in drafting the manuscript, while all authors have given final approval of the version to be published.
